# Mild cognitive impairment in novel *SPG11* mutation-related sporadic hereditary spastic paraplegia with thin corpus callosum: case series

**DOI:** 10.1186/s12883-020-02040-4

**Published:** 2021-01-11

**Authors:** Chuan Li, Qi Yan, Feng-ju Duan, Chao Zhao, Zhuo Zhang, Ying Du, Wei Zhang

**Affiliations:** Department of Neurology, Tangdu Hospital, Fourth Military Medical University, Xi’an City, 710038 Shaanxi Province China

**Keywords:** *SPG11*, Next generation sequencing, Hereditary spastic paraplegia, Mild cognitive impairment

## Abstract

**Background:**

*SPG11* mutation-related autosomal recessive hereditary spastic paraplegia with thin corpus callosum (HSP-TCC) is the most common cause in complicated forms of HSP, usually presenting comprehensive mental retardation on early-onset stage preceding spastic paraplegias in childhood. However, there are many instances of sporadic late-onset HSP-TCC cases with a negative family history, and potential mild cognitive deficits in multiple domains may be easily neglected and inaccurately described.

**Methods:**

In this study, we performed next generation sequencing in four sporadic late-onset patients with HSP-TCC, and combined Mini-Mental State Examination (MMSE) and Montreal Cognitive Assessment (MoCA) to evaluate cognition of the patients.

**Results:**

By evolutionary conservation and structural modeling analysis, we have revealed 4 novel pathogenic *SPG11* mutations, and firstly confirmed mild cognitive impairment (MCI) with normal MMSE scores (≥27) and decreased MoCA scores (< 26) in these *SPG11* mutation-related HSP-TCC patients, predominantly presenting impairment of executive function, delayed recall, abstraction and language.

**Conclusions:**

The results expand the mutational spectrum of *SPG11*-associated HSP-TCC from sporadic cases, and confirm MCI with combination of decreased MoCA and normal MMSE assessment, suggesting that clinicians should consider doing a MoCA to detect MCI in patients with HSP, particularly those with HSP-TCC.

## Background

Hereditary spastic paraplegia (HSP) is a heterogeneous group of genetically-driven neurodegenerative disorder, inherited in autosomal dominant (AD), autosomal recessive (AR) or X-linked patterns with onset age varying from infancy to late adulthood, and traditionally classified into pure and complicated forms [1]. Among them, pure HSP is characterized by bladder involvement, weakness in isolation, slowly progressive spasticity and impaired vibration sense in the lower limbs, while complicated HSP is characterized by spastic paraplegias associated with additional neurological features such as cognitive deficits, thinning corpus callosum (TCC), seizures, amyotrophy, ataxia, extrapyramidal disturbance, visual or auditory disturbances, orthopaedic abnormalities and peripheral neuropathy [2]. Up to now, at least 84 different loci and 67 genes have been shown to be associated with HSP. Among these genes, *SPG11* is the most common cause of complicated autosomal recessive hereditary spastic paraplegia with thin corpus callosum (HSP-TCC), which has been reported to be homozygous or compound heterozygous mutations [3].

General cognitive deficits in *SPG11*-related HSP-TCC preceding to spastic paraplegia is usually first noticed in childhood for learning difficulties and progresses insidiously to severe functional disability, diagnosed as mental retardation [4]. However, there are still lots of sporadic late-onset *SPG11*-related HSP-TCC cases without positive family history, and hardly diagnosed by only clinical manifestations and neuroimaging. Moreover, these patients may present spastic paraplegia without cognitive complaints, and potential mild cognitive deficits in multiple domains may be easily neglected and inaccurately described by conventional Mini-Mental State Examination (MMSE) assessment [5–7].

In this study, we performed a combined approach of next generation sequencing, evolutionary conservation and structural modeling analysis to genetically assess 4 sporadic HSP-TCC patients without positive family history and prominent cognitive impairment. The results revealed 1 reported and 4 novel pathogenic *SPG11* mutations. Furthermore, we combined MMSE and Montreal Cognitive Assessment (MoCA) to evaluate cognition of the patients, and firstly confirmed mild cognitive impairment (MCI) with normal MMSE scores (≥27) and decreased MoCA scores (< 26), predominantly presenting impairment of multiple cognitive domains including executive function, delayed recall, abstraction and language, which should be paid more attention by neurologists.

## Methods

### Patients and clinical assessments

Four cases with sporadic HSP were recruited. The clinical assessments were approved by the Expert Committee (equal to the Institutional Review Board) of the Tangdu Hospital of Fourth Military Medical University (China), and we have obtained the written informed consents from all the patients and their family members. The patients and their relatives were all Chinese.

Except positive family history and prominent cognitive impairment in complaints, the characteristics in our patients were consistent with the clinical and radiological criteria for the complicated form of HSP-TCC reported in Japan [8], and Italy [9]. The diagnosis was determined by at least three experienced neurologists and radiologists. Because of negative family history, all known possible causes of spastic paraplegia were carefully excluded. All the patients were assessed by Spastic Paraplegia Rating Scale (SPRS) for spastic paraplegia assessment [10], brain and spinal cord MRI scan, nerve conduction studies (NCS) and electromyography (EMG).

### Next generation sequencing

The blood samples were collected for genetic analysis from 4 patients and all their relatives with informed consent. Extraction of genomic DNA from the peripheral blood leukocytes was obtained by using a standard protocol. Genomic DNA was isolated from peripheral leukocytes, fragmented into 150–200 bp length with the use of sonication. The DNA fragments were then processed by end-repairing, A-tailing and adaptor ligation, a 4-cycle pre-capture PCR amplification, and enriched by a custom-designed panel capturing the coding exons of 39 genes associated with spastic paraplegia, including *SPG11*. Paired-end sequencing (150 bp) was performed on Illumina HiSeq X-ten platform to provide a mean sequence coverage of more than 100×, with more than 95% of the target bases having at least 20× coverage.

Raw data was processed by the Illumina pipeline (version 1.3.4) for image analysis, error estimation, base calling and generating the primary sequence data. For the quality control, the Cutadapt (https://pypi.python.org/pypi/cutadapt) and FastQC (www.bioinformatics.babraham.ac.uk/projects/fastqc/) were used to remove 3′−/5′- adapters and low quality reads, respectively. The clean reads were mapped to the human reference genome (UCSC hg19) with the use of the BWA (version 0.7.10, http://bio-bwa.sourceforge.net) [11], duplicate sequence reads were removed by Picard (version 1.85; http://picard.sourceforge.net), and GATK (version 3.1, https://software.broadinstitute.org/gatk/) [12], was used to detect variants. Variants were annotated by ANNOVAR software (version 2015Dec14, http://www.openbioinformatics.org/annovar/) [13], which including function implication (gene region, functional effect, mRNA GenBank accession number, amino acid change, cytoband, etc.) and allele frequency in dbSNP138, 1000 Genomes (Phase3 - Variant Frequencies 5b) and ExAc (exac.broadinstitute.org/) [14], referring to transcriptNM_025137. Damaging missense mutations were predicted by SIFT (sift.bii.a-star.edu.sg/) and PolyPhen-2 (genetics.bwh.harvard.edu/pph2/). Interpretation of the variants according to the American College of Medical Genetics and Genomics (ACMG) recommended standards [15], and all the variants will be categorized into pathogenic, likely pathogenic, uncertain significance (VUS), likely benign and benign.

Sanger sequencing was performed to validate the putative pathogenic variants, allowing segregation analyses where possible. Genetic information of healthy Chinese obtained from local Chinese Millionome Database (CMDB) was identified as healthy controls.

### Structural and functional analysis

*SPG11* protein sequence was obtained from the uniprot database (https://www.uniprot.org/uniprot/Q96JI7). Conserved domain database (CDD) version 3.18 [16] was used to detect conserved structure domain in *SPG11* via RPS-blast with position-specific score matries (PSSMs), Expect Value threshold was set to 0.01.

Polyphen2 was used to predict possible structural or functional impact of amino acid substitution detected in human proteins using physical and evolutionary comparative algorithm, default setting was used. The prediction was against a precomputed database comprising ~ 150 million missense SNPs detected in all exons of UCSC human genome (hg19) [17].

The orthologous genes of *SPG11* were detected via blast, and the evolution tree was drawn by the gene orthology/paralogy predition method implemented in the ensemble database (http://asia.ensembl.org). Multiple sequence alignment was made using muscle 3.8 [18], Green bars shows areas of conserved peptides in the sequence, white areas are gaps in the alignment. The multiple alignment sequence used to draw the seqlogo figure using the weblogo software [19] (http://weblogo.berkeley.edu/). The sequence logo consists of stacks of symbols for corresponding amino acids. The height of the stack indicates the conservation of amino acids. Chemical properties of amino acids were used to define the color system: polar amino acids (G, S, T, Y, C, Q, N) are green, basic (K, R, H) blue, acidic (D, E) red and hydrophobic (A, V, L, I, P, W, F, M) amino acids are black.

De novo 3D structure modeling was performed using the I-TASSER algorithm [20] for both wild and mutant *SPG11*, It identified first 10 possible structure templates using a meta-server threading approach LOMETS [21] based on the highest significance Z-score of the threading alignments, and used SPICKER program to select the final simulation model based on pair-wise structure similarity using RMSD (TM-score). The confidence of the model is quantitatively measured by C-score. The first model with the best C-score is selected for further analysis.

Simulated protein 3D structures of wild and mutant *SPG11* were aligned using superpose version 1.0 [22]. Protein super positions were calculated using a quaternion approach. Rasmol version 2.7 [23] was used to visualize the wild and mutant structures. Relative position of mutation site was determined using structure alignments.

### Neuropsychological evaluation

All the patients were assessed by Neuropsychiatric Inventory (NPI), MMSE and MoCA for neuropsychological assessments. The NPI and MMSE were administered at the beginning, followed by MoCA on 7th hospital day to avoid effects of habituation. A cutoff of ≥27 on the MMSE was chosen to indicate normal cognitive function and the accepted cutoff of < 26 on the MoCA was taken to indicate cognitive impairment [24, 25]. A cutoff of ≥26 on the ADL was chosen to indicate functional disability.

MoCA is a 30-point test administered in 10 minconsisting of seven subtests. Executive functions are assessed using a clock-drawing task (3 points), a three-dimensional cube copy (1point) and the Trail Making B task (1 point). Naming is assessed using a three-item confrontation naming task with low-familiarity animals (lion, camel, rhinoceros; 3 points). Attention is evaluated using a sustained attention task (target detection using tapping; 1 point), a serial subtraction task (3 points) and digits forward and backward (1 point each). Language is assessed using repetition of two syntactically complex sentences (2 points) and a phonemic fluency task (1 point). Abstraction is assessed using a two-item verbal abstraction task (2 points). The short-term memory recall task (5 points) involves two learning trials of five nouns and delayed recall after approximately 5 min. Finally, orientation to time and place is evaluated (6 points) [25].

## Results

### Clinical features

**Fig. 1:**
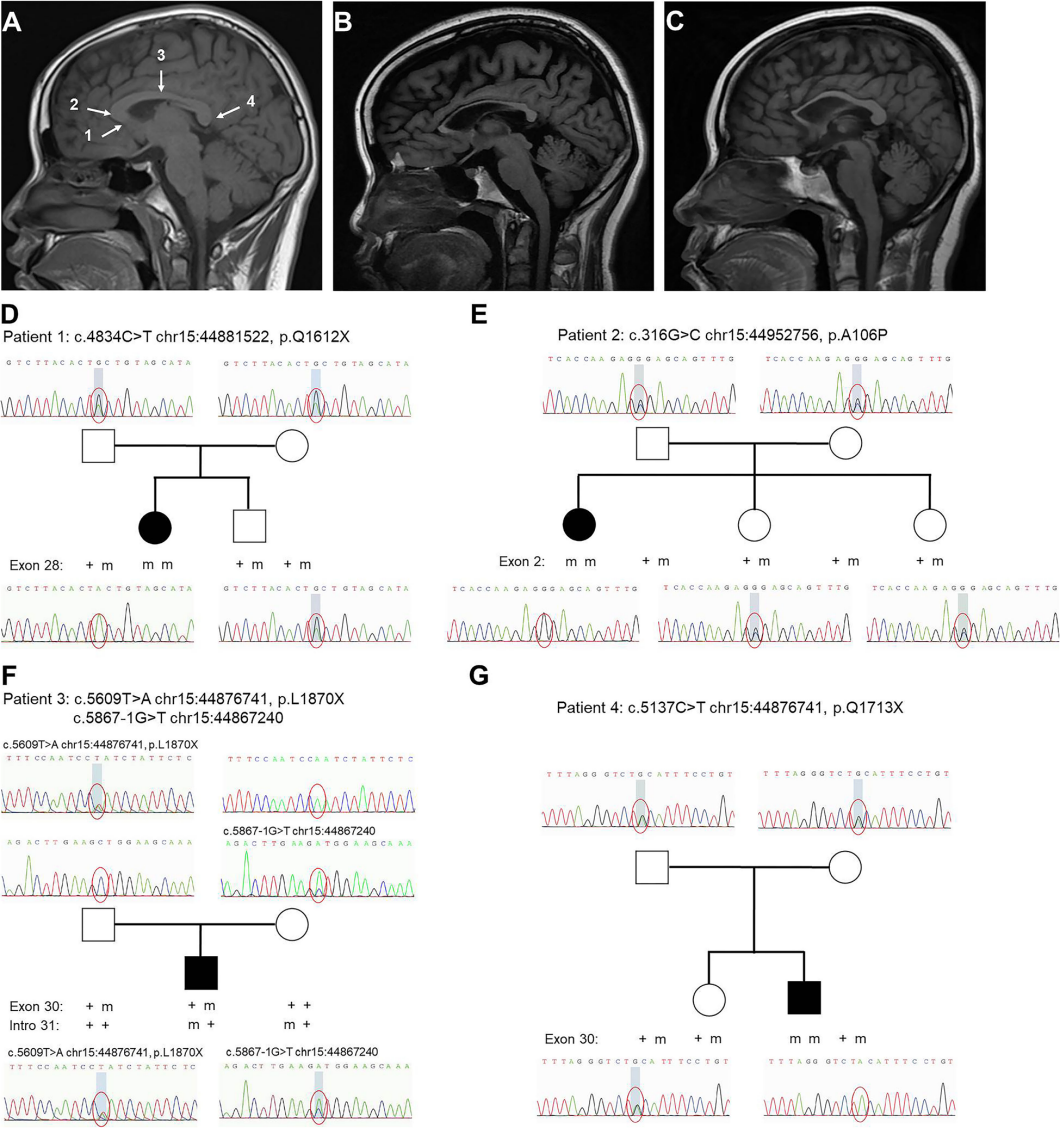
Presentations of corpus callosum in normal and *SPG11*-related HSP-TCC patients in midline sagittal brain MRI. **a** T1-weighted MR image shows normal corpus callosum anatomy in a 24-year-old female (1: rostrum, 2: genu, 3: body, 4: splenium). T1-weighted MR images of patient 1 (**b**) and patient 2 (**c**) with *SPG11* homozygous mutation show thinning of whole corpus callosum most pronounced in the genu and body parts, relatively spared in the splenium. Pedigrees and mutation segregation of the families with *SPG11* mutations: patient 1 (**d**), patient 2 (**e**), patient 3 (**f**), and patient 4 (**g**). The squares indicate male; circles indicate female; filled shape, affected. M: mutation; +: wild type respectively. Chromatograms showing the mutations and respective wild type sequences are shown below the pedigrees

Four sporadic spastic paraplegias patients (2 male and 2 female) and their close relatives were studied. Ages at onset were based on information provided by the patients, the median age at onset for motor symptoms was 23 years (ranging from 18 to 27 years), and the median course of disease was 4 years (ranging from 3 to 7 years). The symptoms at onset were all spasticity, and the spastic paraplegia was evaluated by Spastic Paraplegia Rating Scale (SPRS). Furthermore, no dysarthria, dysphagia, skeletal deformity, cerebellar signs, ophthalmoplegia, decreased vision, sphincter disturbance, amyotrophy, extrapyramidal signs, epilepsy, cataract, or optic atrophy was found in each patient. All patients showed normal in both NCS and EMG examinations. The clinical features of patients were listed in Table 1. Brain MRI showed thinning of whole corpus callosumin all patients, mostpronounced in the genu and body parts, relatively spared in the splenium, without obvious periventricular white matter changes or cortical atrophy, while whole spinal cord MRI of each patient was normal (Fig. 1a-c).

**Table 1:** Summary of clinical presentations and novel mutations in sporadic *SPG11*-related HSP patients in this study

	Patient 1	Patient 2	Patient 3		Patient 4
**Inheritance**	Sporadic	Sporadic	Sporadic		Sporadic
**Sex**	F	F	M		M
**Schooling years**	14	16	15		14
**Initial symptoms**	Spasticity	Spasticity	Spasticity		Spasticity
**UL reflexes**	++	++	++		++
**LL reflexes**	++++	++++	+++		+++
**SPRS**	9	10	7		7
**NPI**	0	0	0		0
**MMSE**	30/30	30/30	29/30		28/30
**MoCA**	22/30	18/30	16/30		17/30
Executive function	2/5	1/5	1/5		2/5
Delayed recall	2/5	2/5	1/5		1/5
Abstraction	1/2	0/2	0/2		0/2
Language	2/3	1/3	0/3		0/3
Naming	3/3	3/3	3/3		3/3
Attention	6/6	5/6	5/6		6/6
Orientation	6/6	6/6	6/6		5/6
**MRI**					
TCC	+	+	+		+
PWM changes	–	–	–		–
Cortical atrophy	–	–	–		–
Ventricular Dilation	–	–	–		–
Cerebellar atrophy	–	–	–		–
Spinal cord	–	–	–		–
**Novel mutations**					
Inheritance	Homozygous	Homozygous	Compound heterozygous		Homozygous
Location	exon 28	exon 2	exon 30	intro 31	exon 30
Mutation	c.4834C > T chr15:44881522	c.316G > C chr15:44952756	c.5609 T > A chr15:44876741	c.5867-1G > T chr15:44867240	c.5137C > T chr15:44876741
Consequence	Stop codon	Missense mutation	Stop codon	Frameshift mutation	Stop codon
Effect	p.Q1612X	p.A106P	p.L1870X		p.Q1713X
ACMG criteria	PVS1 + PM2 + PM3 (pathogenic)	PM2 + PM3 (VUS)	PVS1 + PM2 + PM3 (pathogenic)	PVS1 + PM2 + PM3 (pathogenic)	PVS1 + PM2 + PM3 (pathogenic)

*M* Male, *F* Female, *UL* Upper limbs, *LL* Lower limbs, *SPRS* Spastic Paraplegia Rating Scale, *NPI* Neuropsychiatric Inventory, *MMSE* Mini Mental State Examination, *MoCA* Montreal Cognitive Assessment, *nd* Not done, *MRI* Magnetic resonance imaging, *TCC* Thin corpus callosum, *PWM* Periventricular white matter, + Presence;– Absence, *ACMG* The American College of Medical Genetics and Genomics recommended standards, *VUS* Variants of uncertain significance

### Genetic findings

In this study, we performed a custom-designed panel sequencing by next-generation sequencing in four sporadic patients with spastic paraplegia and TCC, and identified novel as well as reported *SPG11* mutations. Five mutations were recognized, and fourof them, including 3 stop codon (c.4834C > T chr15:44881522/p.Q1612X, c.5137C > T chr15:44876741/p.Q1713X, c.5609 T > A chr15:44876741/p.L1870X) and 1 missense mutation (c.316G > C chr15:44952756/p.A106P), were identified for the first time. (Table 1). The mutations were homozygous in 3 patients, and compound heterozygous in 1 patient. Results of Sanger sequencing indicated that these mutations segregated with the disease in patients while healthy parents and siblings of patients were all asymptomatic heterozygous carriers (Fig. 1d-g). Copy number variants (CNVs) of genes listed in the panel were also tested, and no associated CNVs were identified.

From patient 1, a novel homozygous stop coding mutation was detected in exon28 (c.4834C > T) of *SPG11*, predicted to truncate the functional protein (PVS1). This mutation was never reported in public genomic databases (ExAC or gnomAD), nor detected in healthy controls (PM2). According to ACMG criteria, this mutation was classified to be likely pathogenic (PVS1 + PM2).

The *SPG11* gene of patient 2 displayed a novel homozygous missense c.316G > C (p.A106P) in exon 2. This missense was not identified in ExAC, gnomAD, or healthy controls (PM2). Some in silico algorithms, including SIFT and PolyPhen_2, performed the prediction of pathogenicity of this mutation. This mutation was graded as VUS based on the standard of ACMG. However, considering the absence of other variation or CNV in HSP related gene of this HSP-TCC patient, it is highly possible that this missense was the associated disease-causing loci.

The sequencing result indicated heterozygous mutations that c.5609 T > A (p.L1870X) and c.5867-1G > T presented in two alleles of the *SPG11* gene respectively in patient 3. c.5609 T > A was predicted to prematurely truncate the protein product (PVS1) and was neither reported in public genomic databases nor detected in healthy controls (PM2). It’s in *trans* mutation, mutation c.5867-1G > T, was previously published as Pathogenic (rs1060501168) (PM3) [26]. Thus mutationc.5609 T > A was classified as Pathogenic (PVS1 + PM2 + PM3) according to ACMG criteria.

In patient 4, the novel homozygous nonsense was predicted to truncate the *SPG11* in exon 30(c.5137C > T) (PVS1). This mutation was never reported in ExAC or gnom AD and was not detected in controls (PM2). Two patients were identified in this family with genotypes co-segregated with the disease (PP1). Therefore, this mutation was classified as pathogenic (PVS1 + PM2 + PP1) through ACMG standards.

To accept the variants found via NGS, we have preformed the sanger sequencing test. All 4 mutations were validated and confirmed.

### Pathogenicity of the mutations

**Fig. 2:**
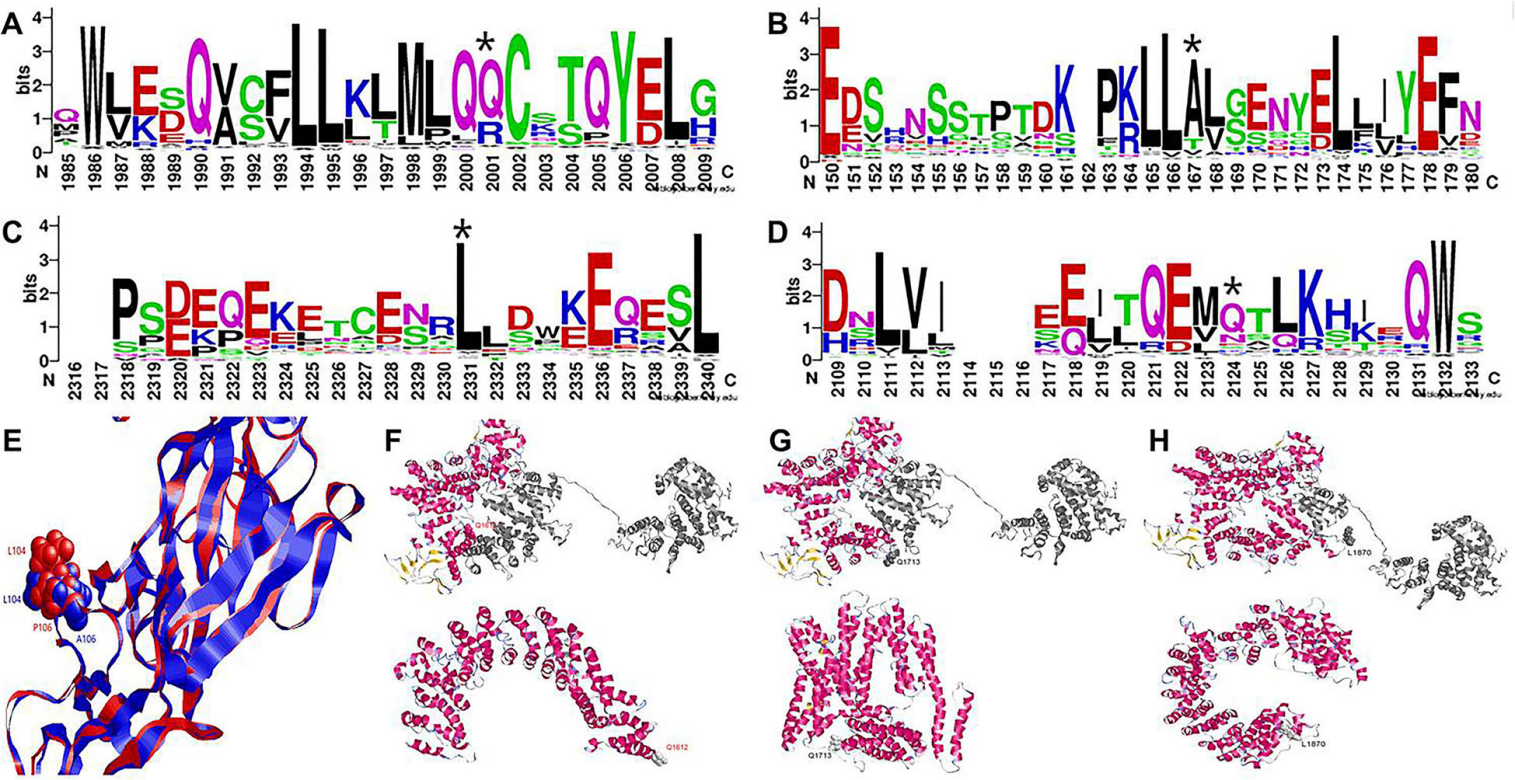
Evolutionary and structural modeling analysis of 4 SPG11 mutants. **a** In patient 1, the mutation site (Q1612X) was located at the 2001amino acid position, and Glutamine was the most conserved. **b** In patient 2, the mutation site (A106P). was located at the 167amino acid position, Alanine was the most conserved residue, while Proline mutation was not presented in the evolution. **c** In patient 3, the mutation site (L1870X) was located at the 2331 amino acid position, where Leucine was highly conserved. **d** In patient 4, the mutation site (Q1713X) was located at the 2124amino acid position and Glutamine was the most conserved. **e** Structural modeling analysis showed that the mutation of A106P in patient 2 caused a sufficient surface exposure and orientation change of L104, and made the nearby peptide SRNSSTPTEKPKL (92–104) to be a potential epitope. Structural modeling analysis showed that patient 1 (**f**), patient 3 (**g**) and patient 4 (**h**) were all stop gain mutations, causing the lost of the helix richc-terminal part of the SPG11, which could be important for structural stabilization

Conservation of these 4 mutations were calculated based on multiple sequence alignment of 141 *SPG11* homologous sequences. All these 4 residues were evolutionarly conserved in over 50% orthologous. In patient 1, the mutation site (Q1612X) was located at the 2001amino acid position where the main variants were Q and R. Q is the most conserved, suggesting the importance of Q. The stop gain mutation at this site may lead to the pathogenicity. In patient 2, the mutation site (A106P) was located at the 167 amino acid position in the below seqlogo, where A,T and V were common residues occurred in evolution across variants species. Alanine was the most conserved residue, however, Polin was rare. The Proline mutation was not presented in the evolution. In patient 3, the mutation site (L1870X) was located at the 2124 amino acid position where the main mutations was L, suggesting the importance of L at this position. The stop gain mutation may lead to the pathogenicity. In patient 4, the mutation site (Q1713X) was located at the 2124 amino acid position and the main variants were Q and N, and Q was the most conserved. The stop gain mutation may lead to the pathogenicity. (Fig. 2a-d).

Among them, A106P in patient 2 was predicted to be probably damaging with polyphen-2 score 0.98 (sensitivity 0.76, specificity 0.96), and the other 3 mutations in patient 1, 3 and 4 were all stop gains. To better understanding the role of these variants, structural modeling analysis was performed. The prediction with the Bepipred Linear Epitope Prediction 2.0 (http://www.cbs.dtu.dk/services/BepiPred/cite.php) showed that the mutation of A106P caused a sufficient surface exposure and orientation change of L104, and made the nearby peptide SRNSSTPTEKPKL (92–104) to be a potential epitope, The other 3 variants were all stop gain mutations, causing the lost of the helix richc-terminal part of the SPG11, which could be important for structural stabilization.(Fig. 2e-h). Taking together, it seems that the c-terminal domain of SPG11 could be an important binding domain with other proteins and it is stabilized with a helix rich structure to the core region of SPG11. All these 3 variants (Q1612X, Q1713X, L1870X) introduced stop codon to the beginning position of the binding domain, thereby causing damages to both the SPG11 core structure and potential protein interaction function.

### Neuropsychological findings

All the patients have been educated for more than 12 years. NPI was performed in them for psychiatric assessment, and the results showed normal. MMSE was performed in these patients for preliminary cognitive assessment, and the score of each patient was normal ranging from 28 to 30, according to accepted normal MMSE cutoff value ≥27, suggesting no general cognitive decline or dementia. Moreover, all the patients were reevaluated by MoCA in detail, and the score of each patient was decreased ranging from 16 to 22, according to accepted abnormal cutoff value < 26. Among the seven subtests of MoCA, the average score of “Executive functions (5 points)” was 1.5 (ranging from 1 to 2); the average score of “Delayed recall (5 points)” was 1.5 (ranging from 1 to 2); the average score of “Abstraction (2 points)” was 0,25 (ranging from 0 to 1); the average score of “Language (3 points)” was 1 (ranging from 0 to 2); the average score of “Naming (3 points)” was 3; the average score of “Attention (6 points)” was 5.5 (ranging from 5 to 6); the average score of “Orientation (6 points)” was 5.75 (ranging from 5 to 6) (Table 1). The results of MoCA showed potential impairments in multiple cognitive domains that are not detected by the MMSE, including executive function, delayed recall, abstraction and language. Altogether, combination of MMSE and MoCA confirmed mild cognitive impairment (MCI) in these sporadic *SPG11*-related HSP-TCC cases.

## Discussion

In present study, we have reported four sporadic HSP-TCC cases, and found novel *SPG11* mutations in each patient by next generation sequencing. Among them, truncating variants included two homozygous stop-gain mutations in patient 1 and 4, and one compound heterozygous stop-gain and frameshift mutation in patient 3, while homozygous missense mutation in patient 4 belonged to non-truncating variant. Moreover, systematical evaluation showed that all 4 variants occur at evolutionary conserved residues, and present highly possible damages for either structural stabilization or potential protein binding ability of SPG11, suggesting pathogenenicity of these mutations. *SPG11* gene encodes endogenous expression of spatacsin, distributed in the neurons forming the corticospinal tract and corpus callosum, as well as in hippocampus, cerebellum and dentate nucleus in adult CNS [27]. Loss of function may affect axonal transport and autolysosomes accumulation, contributing to diffuse neurodegeneration and causing clinical heterogeneity (presenting ataxia, parkinsonism or cognitive impairment in addition to core feature of spastic gait) in *SPG11*-related HSP-TCC [28, 29]. Among these complicated symptoms, non-movement disorders, such as cognitive profile and subtle deficits, may be easily ignored or inaccurately described. In previous retrospective studies about *SPG11*-related HSP-TCC, only 19% (4/21) patients has been reported cognitive impairments at onset, and almost 27% (4/15) patients has described as “TCC without cognitive impairment”. After longitudinal follow-up, cognitive dysfunction clearly worsened with disease progression [27, 30].

The corpus callosum is the main commissural pathway linking the hemispheres of the brain, and identifiable anatomically divided into four parts: rostrum, genu anteriorly, body centrally and splenium posteriorly. Thinning of the corpus callosum (TCC) is a key manifestation and often only imaging feature in early *SPG11*-related familial or sporadic HSP, and almostly can be found in each patient, predominantly thinning in the genu and body, relatively sparing in the splenium of corpus callosum, which is also consistent with four sporadic patients reported in our study. The project fibers arising from prefrontal and motor cortex mainly pass through the genu and body respectively, and further research has also demonstrated that the prefrontal and motor portions of the corpus callosum are preferentially affected in *SPG11*-related HSP-TCC [31–33]. Moreover, voxel-based analysis in neuroimaging also showed global reduction of generalized fractional anisotropy (GFA) in the cerebral white matter innervating cortical regions, including prefrontal, parietal and temporal cortex, suggesting impaired microstructural integrity in fibers and restricted cerebral hypometabolism for diffuse cognitive deficits in *SPG11*-related HSP-TCC [31–33].

Although early and widespread impairment of cognitive functions across all domains is a well-known phenomenon in *SPG11*-related HSP-TCC, there are still some cases of TCC without cognitive impairment as reported previously. Potential mild cognitive deficits in some specific domains may be easily neglected by neurologist’s clinical impression and inaccurately described by conventional MMSE, which is widely used for screening general cognitive decline or dementia, but inadequate for subtle cognitive impairment [5–7]. Unlike MMSE, MoCA is designed to adding assessments or increasing difficulties of tasks such as execution, abstraction, delayed recall and language, and widely used to evaluate mild cognitive impairment (MCI) in degenerative diseases [25]. More recently, a research about cognitive profiles in *SPG4*-related pure HSP has found that subtle cognitive deficits not compatible with dementia were detected by discrepancy between normal MMSE and decreased MoCA scores in adult patients, and there was similar cognitive performances between truncating (nonsense and frameshift mutations) and non-truncating (missense and in-frame insertion) variants, which is consistent with *SPG11*-related HSP-TCC patients reported in our study [25].

MCI is an intermediate clinical state between normal cognitive function and dementia with decline on objective cognitive tasks, and the aetiology of MCI is heterogeneous including different neurodegenerative diseases, ischaemia, trauma, metabolic disturbance, etc. [32]. Neuropsychological testing can be helpful to distinguish MCI from normal or dementia cases, for dementia usually presenting clear impairment in functional activities and scores low on MMSE, while MCI showing particularly subtle deficits by more sensitive measures such as MoCA [34]. In fact, 75% of patients with MCI on neuropsychological testing had normal MMSE but abnormal MoCA, and sensitivity of MoCA for MCI is almost 90–100% [25]. In our study, the combination of MMSE and MoCA has confirmed MCI in *SPG11*-related HSP-TCC, characteristically presenting potential deficits in multiple cognitive domains that were not detected by the MMSE, including executive function and abstraction (not tested by the MMSE) and delayed recall and language repetition (MMSE items too easy). These specific phenotypes of cognitive deficits in our patients were also in agreement with *SPG4*-related pure HSP as described before, suggesting that MoCA is likely to be a more sensitive screening tool for subtle cognitive changes in both pure and complicated HSP.

## Conclusions

Collectively, in present study, we have performed next generation sequencing in four sporadic HSP-TCC patients without cognitive complaints, and revealed 3 homozygous and 1 compound heterozygous novel *SPG11* mutations. In addition, we systematically evaluated the pathogenenicity of these mutations via evolutionary conservation and structural modeling analysis. We found that all 4 variants occur at evolutionary conserved residues, and the mutations were all highly possible damaging for either structural stabilization or potential protein binding ability of SPG11. Our findings expand the mutational spectrum of *SPG11*-associated HSP-TCC from sporadic cases. Moreover, we firstly confirm MCI by combination of MMSE and MoCA in these patients, characteristically presenting potential deficits in multiple cognitive domains (including executive function, delayed recall, abstraction and language) in *SPG11*-related HSP-TCC, which should be paid more attention by neurologists.

## Data Availability

The dataset analysed are available from the corresponding author on reasonable request.

## Abbreviations

MCI: Mild cognitive impairment
HSP: Hereditary spastic paraplegias
AD: Autosomal dominant
AR: Autosomal recessive
MRI: Magnetic resonance imaging
TCC: Thincorpus callosum
MMSE: Mini-Mental State Examination
MoCA: Montreal Cognitive Assessment
SPRS: Spastic ParaplegiaRating Scale
NPI: Neuropsychiatric Inventory
CMDB: Chinese Millionome Database
ACMG: American College of Medical Genetics and Genomics
VUS: Variant of Uncertain Significance
CNVs: Copy Number Variations
